# *Cc2d1b* Contributes to the Regulation of Developmental Myelination in the Central Nervous System

**DOI:** 10.3389/fnmol.2022.881571

**Published:** 2022-05-03

**Authors:** Jenica Acheta, Jiayue Hong, Haley Jeanette, Simrandeep Brar, Anish Yalamanchili, M. Laura Feltri, M. Chiara Manzini, Sophie Belin, Yannick Poitelon

**Affiliations:** ^1^Department of Neuroscience and Experimental Therapeutics, Albany Medical College, Albany, NY, United States; ^2^Departments of Biochemistry and Neurology, Institute for Myelin and Glia Exploration, Jacobs School of Medicine and Biomedical Sciences, State University of New York at Buffalo, Buffalo, NY, United States; ^3^Department of Neuroscience and Cell Biology, Rutgers-Robert Wood Johnson Medical School, Child Health Institute of New Jersey, New Brunswick, NJ, United States

**Keywords:** myelin, Schwann cell, oligodendrocyte, Cc2d1b, myelination

## Abstract

**Background:**

Numerous studies have indicated that myelination is the result of the interplay between extracellular signals and an intricate network of transcription factors. Yet, the identification and characterization of the full repertoire of transcription factors that modulate myelination are still incomplete. CC2D1B is a member of the Lgd/CC2D1 family of proteins highly expressed in myelinating cells in the central and peripheral nervous systems. In addition, the absence of CC2D1B limits myelin formation *in vitro*. Here we propose to delineate the function of CC2D1B in myelinating cells during developmental myelination *in vivo* in the central and peripheral nervous systems.

**Methods:**

We used a *Cc2d1b* constitutive knockout mouse model and then performed morphological analyses on semithin sections of sciatic nerves and electron micrographs of optic nerves. We also performed immunohistological studies on coronal brain sections. All analyses were performed at 30 days of age.

**Results:**

In the peripheral nervous system, animals ablated for *Cc2d1b* did not show any myelin thickness difference compared to control animals. In the central nervous system, immunohistological studies did not show any difference in the number of oligodendrocytes or the level of myelin proteins in the cortex, corpus callosum, and striatum. However, optic nerves showed a hypomyelination (0.844 ± 0.022) compared to control animals (0.832 ± 0.016) of large diameter myelinated fibers.

**Conclusions:**

We found that CC2D1B plays a role in developmental myelination in the central nervous system. These results suggest that CC2D1B could contribute to gene regulation during oligodendrocytes myelination in optic nerves.

## Introduction

Oligodendrocytes and Schwann cells are myelinating cells in the central nervous system (CNS) and peripheral nervous system (PNS), with a similar role in producing myelin to insulate axons and facilitate fast propagation of action potential. While oligodendrocytes and Schwann cells differ on many levels, myelin formation in both cell types is the result of the integration of biochemical signaling pathways and mechanical stimuli coming from the extracellular matrix, and neighboring cells—including the axon. These signals regulate an intricate network of transcription factors and epigenetic programs that control the proliferation, migration, differentiation, and maturation of myelinating cells. The identification and characterization of the complete repertoire of transcription factors that modulate myelination are still incomplete (Emery and Lu, 2015; Sock and Wegner, 2019).

*Cc2d1b*, also named *Freud-2* or lethal (2) giant discs-1, encodes for Coiled-coil and C2 domain containing 1B protein. CC2D1B and its homolog CC2D1A are thought to have redundant functions as they contain very similar protein domains. They contain a C2 domain, which allows binding to membrane lipids (Gallagher and Knoblich, 2006; Drusenheimer et al., 2015), and four DM14 domains, which allow interaction with endosomal sorting complex ESCRT-III (Drusenheimer et al., 2015; Ventimiglia et al., 2018). In addition, CC2D1A and CC2D1B have been reported to regulate gene transcription, as they both can bind a dual repressor element in the HTR1A promoter and repress the expression of serotonin 1A receptor in neurons (Hadjighassem et al., 2009, 2011). However, while *Cc2d1a* is highly expressed in neurons and has been implicated in intellectual disability and autism spectrum disorder (Basel-Vanagaite et al., 2006; Manzini et al., 2014), *Cc2d1b* has been associated with myelin formation (Belin et al., 2019). We and others have previously shown that *Cc2d1b* is expressed in mouse sciatic nerves and more specifically in myelinating and non-myelinating Schwann cells (Belin et al., 2019; Gerber et al., 2021). In addition, Schwann cell-knockdown of *Cc2d1b* in Schwann cell-neuron cocultures impairs myelination *in vitro* independently of effects on Schwann cell number, proliferation, or apoptosis (Belin et al., 2019). *Cc2d1b* knockout (KO) mice have been reported and present delayed memory acquisition and retention (Zamarbide et al., 2018). The growing evidence, from both animal studies and human neuroimaging suggests that myelin plays a role in learning (McKenzie et al., 2014; Sampaio-Baptista and Johansen-Berg, 2017; Bacmeister et al., 2020), therefore the role of CC2D1B in the CNS must be considered.

Here we show that in *Cc2d1b* KO mice while CC2D1B appears dispensable for PNS radial myelination, CC2D1B is required for proper developmental myelination of large diameters (>1.5 μm) myelinated fibers in the optic nerves. These results suggest that CC2D1B could contribute to gene regulation during oligodendrocytes myelination in the optic nerves.

## Materials and Methods

### Animal Model

All experiments involving animals followed experimental protocols approved by the Albany Medical College Institutional Animal Care and Use Committee. *Cc2d1b* KO mice (Zamarbide et al., 2018) were derived from a congenic C57BL/6J background (as described in Poitelon et al., 2016). Genotyping of mutant mice was performed by PCR on tail genomic DNA [as described by Zamarbide et al. (2018)]. Animals were housed in cages of five in 12/12 h light/dark cycles. Mice were all housed with sex-matched littermates following weaning. All mice were given ad libitum access to food and water. No animals were excluded from the study. For this study, heterozygous parents were bred to obtain both KO and WT mice and both male and female mice were used. Mutant and control littermates were sacrificed at the indicated ages, and sciatic, optic nerves, and brains were collected. This study was carried out by the recommendations of ARRIVE guidelines and approved by the Albany Medical College Institutional Animal Care and Use Committee (no. 20-08001).

### Western Blotting

Right after sampling sciatic nerves were flash-frozen in liquid nitrogen, pulverized, and resuspended in lysis buffer 95 mM NaCl, 25 mMTris-HCl pH 7.4 10 mMEDTA, 2% SDS, 1% Protease Inhibitor Cocktail (Roche Diagnostic), 1% phosphatase inhibitor cocktail 2 and 3 (Sigma Aldrich, P5726 and P0044). Protein lysates were centrifuged at 15,000 *g* for 30 min at 4°C. Supernatant protein concentrations were determined by bicinchoninic acid assay protein assay according to the manufacturer’s instructions. Equal amounts of homogenates were diluted 3:1 in 4× Laemmli (250 mm Tris-HCl, pH 6.8, 8% sodium dodecyl sulfate, 8% β-Mercaptoethanol, 40% Glycerol, 0.02% Bromophenol Blue), denatured 5 min at 100°C, resolved on SDS-polyacrylamide gel and electro-blotted onto PVDF membrane. Blots were then blocked with 5% bovine serum albumin in 1× Phosphate-buffered saline (PBS), 0.05% Tween-20 and incubated overnight with the following appropriate antibodies: anti-CC2D1A 1/500 (Abcam, ab68302), anti-CC2D1B 1/500 (Proteintech, 20774-1-AP), and anti-Calnexin 1/3,000 (Sigma Aldrich, C4731). Calnexin was used as a standard loading control to normalize protein levels, as CC2D1B (140 KDa) precluded the utilization of GAPDH, Actin, or Tubulin (all <55 kDa). Membranes were then rinsed in 1× PBS and incubated for 1 h with HRP-conjugated secondary antibodies. Blots were developed using ECL or ECL Plus (GE Healthcare). Western blots were quantified using Image J software^1^.

### Morphological Analysis

Mutant and control littermates were euthanized at the indicated ages, and optic nerves and sciatic nerves were dissected. Nerves were fixed in 2% buffered glutaraldehyde for at least 24 h before being postfixed in 1% osmium tetroxide. After alcohol dehydration, samples were embedded in EPON resin. For semithin sections (1 μm thick), samples were stained with toluidine blue and examined by light microscopy. For ultrathin sections (80–85 nm thick), samples were stained with uranile acetate and lead citrate and examined by electron microscopy. For g ratio analysis (axon diameter/fiber diameter), images were acquired with a 100× objective. G ratios were determined for 100 fibers chosen randomly per animal. For all morphological assessments, at least three animals per genotype were analyzed. Data were analyzed using ImageJ software^1^.

### Electrophysiological Analyses

Animals were analyzed at 60 days of age as described previously (Poitelon et al., 2018; Jeanette et al., 2021). Mice were anesthetized with tribromoethanol, 0.4 mg g^−1^ of body weight, and placed under a heating lamp to avoid hypothermia. Motor conduction velocity and amplitude of the sciatic nerve were obtained with subdermal steel monopolar needle electrodes: a pair of stimulating electrodes was inserted subcutaneously near the nerve at the ankle, then at the sciatic notch, and finally at the paraspinal region at the level of the iliac crest to obtain three distinct sites of stimulation, proximal and distal, along the nerve. Compound motor action potentials were recorded with an active electrode inserted in muscles in the middle of the paw and a reference needle in the skin between the first and second digits. Electrophysiological studies comprising motor and sensory nerve conduction studies were conducted using a Viking Quest electromyography device.

### Immunohistochemistry

All animals were anesthetized with 2.5% trimoboethanol (avertin), injected intraperitoneally, and then perfused with 4% of paraformaldehyde (PFA) in PBS *via* the left ventricle. The brains were post-fixed overnight in 4% of PFA at 4°C. Coronal brain slices 50 μm thick were prepared using a cryostat (Leica Biosystems, CM1950). Free-floating sections were incubated in a blocking solution (5% fetal bovine serum and 2% Triton X-100 in PBS) for 2 h at room temperature and incubated with the primary antibody in an incubation solution (2% fetal bovine serum and 2% Triton X-100 in PBS) for 2 h at room temperature. The following primary antibodies were used in this study: anti-MBP 1/1,000 (Biolegend, 808401), anti-PLP-1 1/400 (Abcam, ab284886), anti-MOG 1/500 (Millipore, MAB5680), anti-OLIG2 1/200 (Sigma Aldrich, AB9610), and anti-KI67 1/300 (Thermofisher, 14-5698-82). Control conditions including only the secondary antibodies were used to verify the staining specificity to the primary antibodies. Sections were then rinsed in PBS and incubated with Alexa 488 and 555-conjugated secondary antibodies for 2 h at room temperature. The sections were then rinsed with PBS followed by a counterstain with DAPI. After washing, the sections were mounted on microscopy slides using coverslips and a mounting medium (Vector Laboratories, H-1000). Brain coronal sections images were acquired at a magnification of ×10 Axio ObserverZ1 (Zeiss) equipped with a standard digital camera (exposure time: 8 ms for Dapi, 25 ms for OLIG2, 100 ms for PLP1, 200 ms for MBP, and 250 ms for MOG). Stitching was processed with ZEN 2.3 (Zeiss). The staining intensity for myelin proteins as well as the number of positive cells was assessed in the central area of the corpus callosum, between the midline and below the apex of the cingulum (0.6 mm^2^ area), in the motor cortex including M1, M2 (0.6 mm^2^ area) and the dorsal/caudal striatum, immediately underneath the corpus callosum (0.6 mm^2^ area). The integrated fluorescence intensity was calculated as the product of the area and mean pixel intensity using ImageJ^1^. All quantification of positive cells and fluorescent intensity results were determined from at least four brains per experimental group.

### Statistical Analyses

Experiments were not randomized, but data collection and analysis were performed blind to the conditions of the experiments. Data are presented as mean ± standard error of the mean (s.e.m.). No statistical methods were used to predetermine sample sizes, but our sample sizes are similar to those generally employed in the field. Two-tailed Student’s t-test was used for statistical analysis of the differences between groups. Statistical analyses were performed with Prism 7.0 (GraphPad). Values of *P*-value ≤ 0.05 were considered to represent a significant difference.

## Results

**Figure 1:**
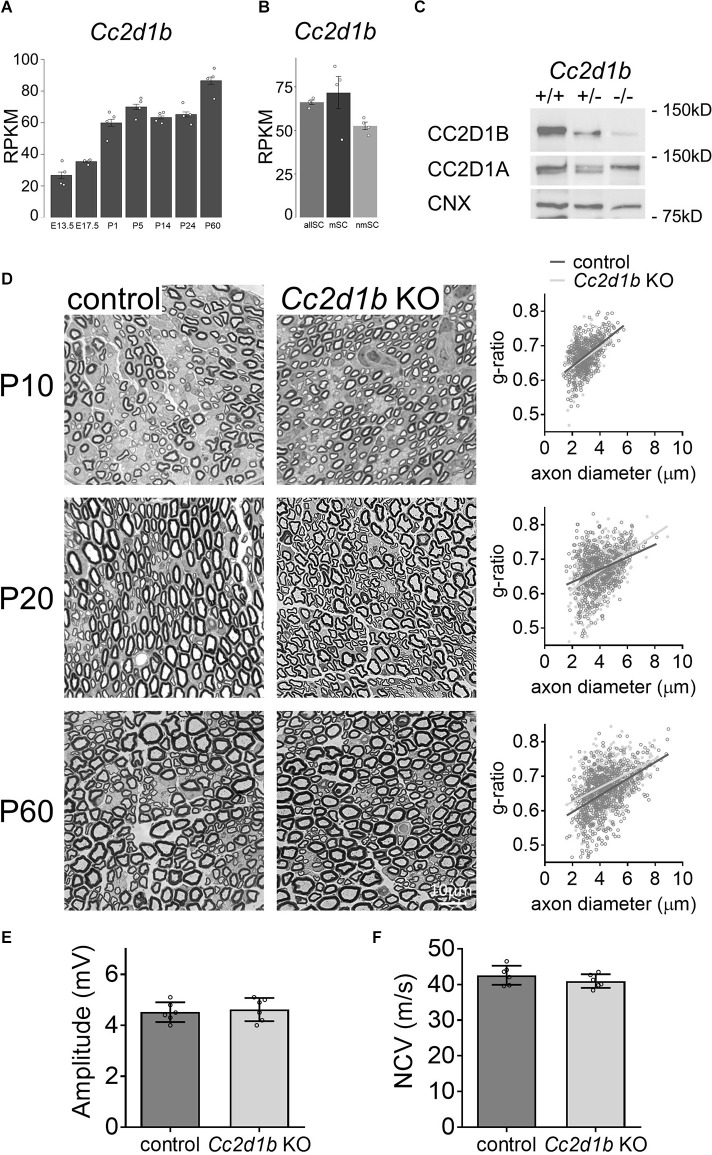
KO of *Cc2d1b* does not alter developmental myelination in the PNS. **(A,B)** Single cells RNA-seq–based gene expression values (RPKM) of *Cc2d1b* in myelinating Schwann cells (mySC), non-myelinating Schwann cells (nmSC), and all Schwann cells (allSC; Gerber et al., 2021). Schwann cells were isolated from mouse sciatic nerve embryonic day 13.5 (E13.5), E17.5, P1, P5, P14, P24, P60 (Gerber et al., 2021). **(C)** Western blot analysis shows that CC2D1B protein levels are decreased in sciatic nerves of *Cc2d1b* KO mice at 30 days of age. CC2D1A protein levels are not affected by the absence of CC2D1B. Calnexin was used as a protein loading control. **(D)** Myelination in *Cc2d1b* KO mice. Semithin analysis of control and *Cc2d1b* KO sciatic nerves at P10, P20, and P60. The thickness of myelin (g ratio) was measured. *n* = 3–5 mice for each genotype. **(E)** Measurements of compound muscle action potential amplitude from *Cc2d1b* KO animals at P60. *n* = 6 nerves for each genotype. **(F)** Measurements of nerve conduction velocity (NCV) from *Cc2d1b* KO animals at P60. *n* = 6 nerves for each genotype. Data are represented as mean ± s.e.m. Scale bars 10 μm.

We and others have previously shown that *Cc2d1b* is expressed by Schwann cells and in sciatic nerves (Belin et al., 2019; Gerber et al., 2021). *Cc2d1b* was notably shown to be expressed by both myelinating and non-myelinating Schwann cells (Gerber et al., 2021; Figure 1A), as well as increased along with sciatic nerve development (Gerber et al., 2021; Figure 1B). To analyze the role of *Cc2d1b* in Schwann cells *in vivo*, we used the constitutive KO for *Cc2d1b*, generated by the Knockout Mouse Project, and previously described (Zamarbide et al., 2018). Similarly, to the previous report, *Cc2d1b* KO mice are viable, fertile, and indistinguishable from wildtype (WT) littermates. However, we did observe a bias in mendelian ratio from the breeding of heterozygote *Cc2d1b* KO, with a WT/heterozygote/KO ratio of 18/64/18 instead of the expected 25/50/25 (*n* = 218, *P*-value ≤ 0.001), for both males and females. We confirmed *via* Western blot analysis using sciatic nerve protein lysates at 30 days of age that CC2D1B was absent in *Cc2d1b* KO and that CC2D1A was expressed at normal levels (Figure 1C).

To investigate the role of CC2D1B in myelin formation, the nerve ultrastructure of*Cc2d1b*KO sciatic nerves was compared with control sciatic nerves by semithin sections. Different developmental stages were analyzed; 10 days of age (P10), when radial sorting in the peripheral nerves is completed, and all immature Schwann cells have differentiated into myelinated or unmyelinated Schwann cells; P20, when myelination is ongoing; and P60, when myelination is completed. At P10, *Cc2d1b* KO axons are properly sorted and the myelin thickness of myelinated fibers in *Cc2d1b* KO sciatic nerves (0.664 ± 0.006) was comparable to control sciatic nerves (0.671 ± 0.005; Figure 1D). Similarly, at P20, no difference in the myelin thickness of myelinated fibers in *Cc2d1b* KO sciatic nerves(0.668 ± 0.012) was observed when compared to control sciatic nerves (0.67 ± 0.01; Figure 1D). At P60, myelin maintenance was not affected in sciatic nerves of *Cc2d1b* KO animals (0.653 ± 0.005) compared to control animals (0.677 ± 0.018; Figure 1D). At P60, we also did not observe any effect on nerve conduction velocity and the amplitude of compound muscle action potentials in *Cc2d1b* KO animals (40.97 m/s ± 0.78; 4.62 mV ± 0.18) compared to control animals (42.62 m/s ± 1.09; 4.52 mV ± 0.16; Figures 1E,F). Overall, these data indicate that CC2D1B is not likely to play a major role in the regulation of radial myelination in the PNS.

**Figure 2:**
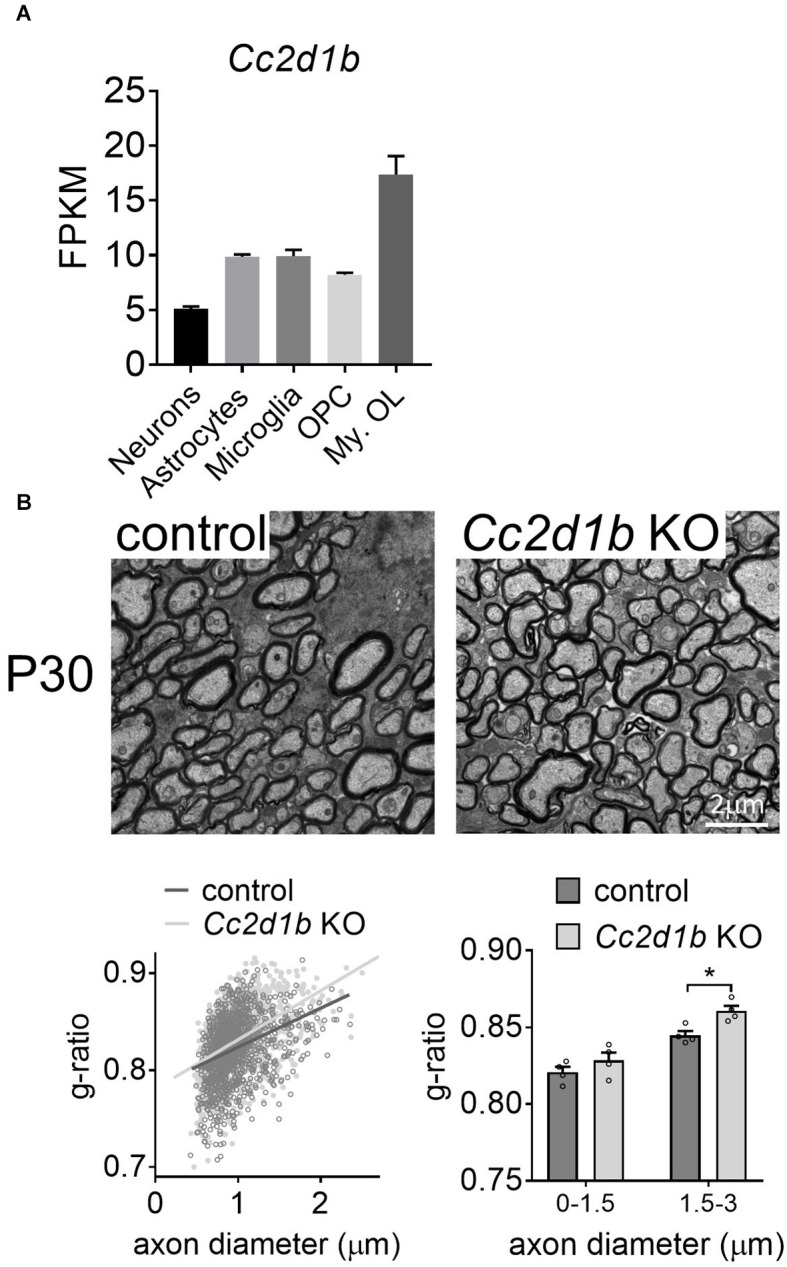
KO of *Cc2d1b* alters myelination of large diameter fibers in the CNS. **(A)** RNA-seq-based gene expression values (FPKM) of *Cc2d1b* in mouse brain neurons, astrocytes, microglia, oligodendrocyte precursor cells (OPC), and myelinating oligodendrocyte (My.OL; Zhang et al., 2014). **(B)** Electron microscopy of the *Cc2d1b* KO optic nerves. (Up) Electron micrographs of axons in the optic nerves of control and *Cc2d1b* KO mice, at 30 days of age. Scale bars, 2 μm. (Down) Scatter plot, and bar graph of g-ratio values of myelinated axons per axon diameter. Mean axonal diameter of myelinated axons. One-hundred fibers per animal were analyzed. *n* = 4 mice for each genotype. Data are presented as mean ± s.e.m. Two-sided Student’s *t*-test: *P-value ≤ 0.05.

**Figure 3:**
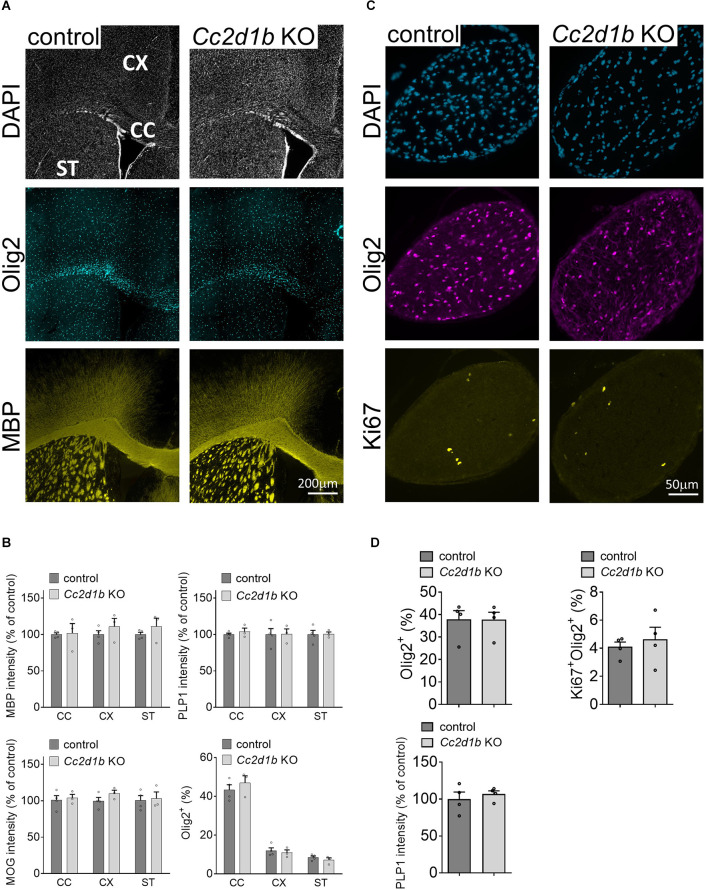
Myelin protein level and OLIG2-positive cell number are not affected in *Cc2d1b* KOCNS. **(A)** Representative coronal sections of brain tissue immunostained for MBP and OLIG2 collected from adult *Cc2d1b* KO animals, at 30 days of age. Scale bar, 200 μm. **(B)** Integrated fluorescence intensity for MBP, MOG, and PLP1 and the number of OLIG2-positive cells were quantified in the lateral corpus callosum (CC), the cingulate cortex (CX), and striatum (ST). **(C)** Representative cross sections of optic nerve immunostained for OLIG2 and KI67 collected from adult *Cc2d1b* KO animals, at 30 days of age. Scale bar, 50 μm. **(D)** Integrated fluorescence intensity for PLP1, and the number of OLIG2-positive and KI67-positive cells were quantified in the optic nerves. *n* = 3–4 mice for genotype. Data are presented as mean ± s.e.m.

CC2D1B was also shown to be expressed and enriched in myelinating oligodendrocytes in the central nervous system (Zhang et al., 2014; Figure 2A). Because *Cc2d1b* KO mice present cognitive deficits (Zamarbide et al., 2018), we sought to clarify the role of CC2D1B in oligodendrocytes during myelin formation. We first investigated optic nerves, a region of the CNS rich in oligodendrocytes and myelin. The degree of myelination of the *Cc2d1b* KO animals was assessed by electron microscopy by calculating the g-ratio of myelinated axons in optic nerves (Figure 2B). Myelinated fibers with a diameter above 1.5 μm (~6% of the total myelinated fiber population) were hypomyelinated in *Cc2d1b* KO (0.844 ± 0.022) compared to control animals (0.832 ± 0.016; *P*-value ≤ 0.05) at P30. The myelination of the *Cc2d1b* KO CNS was also evaluated by immunohistochemistry on brain coronal sections and optic nerves. We measured the level of myelin markers (MBP, MOG, and PLP1) in the cortex, corpus callosum, striatum, and optic nerves, but no significant differences were observed between *Cc2d1b* KO and control CNS structures at P30 (Figure 3). Finally, we also analyzed if the absence of CC2D1B would affect the number of oligodendrocytes. We counted the number of OLIG2 positive cells in the cortex, corpus callosum, striatum, and optic nerves, but did not observe any difference between *Cc2d1b* KO and controls CNS structures at P30 (Figure 3). Overall, these data indicate that CC2D1B may play a role in the regulation of myelin thickness by oligodendrocytes in optic nerves.

## Conclusions/Discussion

We showed that two transcriptional activators YAP and TAZ are essential for myelin formation and regulate DNA-binding protein, *Cc2d1b* (Poitelon et al., 2015; Lopez-Anido et al., 2016; Belin et al., 2019). We also showed that *Cc2d1b* knockdown in Schwann cells impairs myelination *in vitro* (Belin et al., 2019). Here we proposed to delineate the function of this novel regulator during myelination in the PNS and the CNS. We found that while CC2D1B appears to be dispensable for radial myelination in the PNS, our data suggest that CC2D1B could contribute to the regulation of oligodendrocyte myelination in optic nerves during development.

Serotonin receptors are expressed by oligodendrocytes, and *in vitro* serotonin exposure leads to impaired oligodendrocyte differentiation and reduced myelin proteins expression (Fan et al., 2015). Yet, there has been no analysis of the *in vivo* expression of serotonin receptors in oligodendrocytes. CC2D1B was found to be a repressor of serotonin-1A receptor (encoded by *Htr1a*) expression (Hadjighassem et al., 2009, 2011). Our data suggest that the absence of CC2D1B in large diameter fibers could cause an increase in serotonin signaling in oligodendrocytes. Further *in vivo* genetic analysis focused on evaluating serotonin receptors in oligodendrocytes should determine if serotonin is an inhibitor of myelin formation.

In addition, in *Drosophila*, *Cc2d1b*/*Lgd* has been shown to regulate notch signaling through the modulation of intracellular trafficking (Childress et al., 2006; Gallagher and Knoblich, 2006; Jaekel and Klein, 2006). However, in mammals, *Cc2d1b* does not affect notch signaling (Drusenheimer et al., 2015). Instead, *Cc2d1b* was shown: (i) to be involved in the reformation of the nuclear envelope following mitosis, and (ii) to regulate microtubule severance in the mitotic spindle (Ventimiglia et al., 2018; Baeumers et al., 2020). Molecules of similar function, i.e., *Nde1*, *BubR1*, *Tppp*, regulating either microtubule nucleation or the mitotic spindle, have been shown to regulate oligodendrocyte differentiation and myelination (Choi et al., 2016; Shimizu et al., 2018; Fu et al., 2019). Thus, it is also possible that CC2D1B regulates the myelin formation of large diameter fibers through the regulation of the oligodendrocyte cytoskeleton.

CC2D1 proteins, which are structurally very similar, were shown to be both involved in the regulation of MAPK and Toll-like receptor 4 (Deshar et al., 2016). In addition, CC2D1A was shown to be involved in the regulation of multiple signaling pathways, including AKT, NF-κB, protein kinase A (Nakamura et al., 2008; Zhao et al., 2010; Al-Tawashi et al., 2012), which are known to be involved in the regulation of myelination (for review, see Blank and Prinz, 2014; Nave and Werner, 2014). Thus, is also possible that CC2D1B regulates myelin formation in optic nerves through the regulation of essential signaling pathways.

Finally, prior behavioral studies on *Cc2d1b* KO assessing visual cues (visual reach test; Zamarbide et al., 2018) did not detect differences in basic visual function in adult mice. Thus, we should consider that ablation of *Cc2d1b* may delay but not impair the myelin formation of large diameter fibers in optic nerves. In addition, while basic motor and sensory function tests (including visual reach test), object memory, anxiety, and hyperactivity, showed no difference between *Cc2d1b* KO males and females, differences were observed in the Morris Water Maze test, with *Cc2d1b* ablation affecting males’ spatial memory formation and retention but not females (Zamarbide et al., 2018). Thus, it is also possible that subtle sex-specific differences in myelination could be observed in specific regions of the CNS that we did not investigate, such as the hippocampus, which is involved in spatial memory.

## Data Availability Statement

The original contributions presented in the study are included in the article, further inquiries can be directed to the corresponding author/s.

## Ethics Statement

The animal study was reviewed and approved by Albany Medical College Institutional Animal Care and Use Committee (no. 20-08001).

## Author Contributions

SBe and YP: conceptualization, funding acquisition, project administration, validation, investigation, formal analysis, visualization, writing—original draft preparation, and writing—review and editing. JA and JH: writing—review and editing. SBr, AY, and HJ: investigation and formal analysis. MF and MM: resources, writing—review and editing. All authors contributed to the article and approved the submitted version.

## Conflict of Interest

The authors declare that the research was conducted in the absence of any commercial or financial relationships that could be construed as a potential conflict of interest.

## Publisher’s Note

All claims expressed in this article are solely those of the authors and do not necessarily represent those of their affiliated organizations, or those of the publisher, the editors and the reviewers. Any product that may be evaluated in this article, or claim that may be made by its manufacturer, is not guaranteed or endorsed by the publisher.

## Footnotes

^1^ http://imagej.nih.gov/ij
